# Transcriptional predictors of rescue behaviour in ants

**DOI:** 10.1242/jeb.252086

**Published:** 2026-07-17

**Authors:** Luisa Maria Jaimes-Nino, Adi Bar, Inon Scharf, Susanne Foitzik

**Affiliations:** ^1^ Institute of Organismic and Molecular Evolution, Johannes Gutenberg University Mainz, 55128 Mainz, Germany; ^2^ School of Zoology, George S Wise Faculty of Life Sciences, Tel Aviv University, Tel Aviv 6997801, Israel

**Keywords:** Altruistic behaviour, Brain activity, Gene expression, Helping behaviour, Juvenile hormone

## Abstract

Some social animals can recognize and respond to the distress of group members. While well documented in mammals, such behaviour has also independently evolved in ants. To uncover the molecular basis of this social trait, we compared gene expression in the central and peripheral nervous system of *Cataglyphis nigra* workers differing in rescue propensity. RNA-seq of mushroom bodies, optic lobes, central brain and antennae revealed nine genes upregulated across all tissues of rescuers, including those encoding cytochrome P450-9e2 (*CYP9E2*) and arginase, and immune-related genes such as the defensin (*DEFA*) and serine protease snake (*snk*) genes. The mushroom bodies showed the strongest signal, including genes involved in juvenile hormone signalling, polyamine synthesis and immune function. These results suggest that the immune pathways and polyamine metabolism modulate social responsiveness or alarm-cue detection in ants. In contrast, morphological and physiological traits did not differ between rescuers and non-rescuers, indicating that rescue behaviour propensity is governed by the activity of the nervous system.

## Teaser

**Highlighted Article:** Rescue behaviour in the ant *Cataglyphis nigra* is associated with mushroom body neuronal gene activity rather than workers' physical traits. Rescuers upregulate immune and metabolic genes across tissues.

## INTRODUCTION

Rescue behaviour, assistance directed toward an endangered conspecific, is rarely observed in the wild. This helping behaviour is defined by four criteria: the individual in need faces immediate danger; the helper's response is context appropriate; the helper must incur a cost or risk; and the behaviour provides no direct benefit to the helper (Hollis and Nowbahari, 2013; Miler and Turza, 2021). Rescue behaviour costs time and energy, exposes the rescuer to predation, and can delay food detection (Bar et al., 2024; Kwapich et al., 2019; Taylor et al., 2013). It may, however, confer inclusive fitness benefits by protecting related group members from predators and aiding injured individuals (Frank et al., 2018; Kwapich et al., 2019). To date, rescue behaviour has been reported in primates (Jack et al., 2020), humpback whales (Pitman et al., 2017) and ants (Bar et al., 2023; Frank et al., 2018; Miler et al., 2017a; Nowbahari et al., 2009).

In ants, rescue behaviour is a good example of a kin-selected behaviour, in which an individual places itself at risk to aid a trapped relative (Hollis and Nowbahari, 2013). This behaviour, found so far in 25 species of ants according to a recent review (Miler and Turza, 2021), is well developed in some species, such as several *Cataglyphis* and *Formica* species, and the termite-eating Matabele ant *Megaponera analis* (Bar et al., 2023; Frank et al., 2018; Miler et al., 2017a; Nowbahari et al., 2009). Rescue involves complex behaviours, such as digging, pulling appendages and biting restraining objects (for a detailed description, see Szczuka et al., 2024). Rescue behaviour has evolved independently in different ecological contexts, where there is a high risk of entrapment or injury from predators, such as antlions, spiders or dangerous prey, or in species inhabiting sandy habitats, where ants can become trapped by collapsing soil (Hollis, 2017; Hollis and Nowbahari, 2022; Miler and Turza, 2021).

Rescue behaviour is context dependent and not expressed uniformly across all individuals of a group. Ants selectively engage in rescue, typically aiding particular nestmates and responding to distress signals only under specific conditions. For example, rescue behaviour in *Cataglyphis piliscapa* (formerly *C. cursor*; Doums et al., 2023) is triggered only when victims are nestmates and mobile, compared with individuals artificially immobilized by cooling (Nowbahari et al., 2009). In other species, such as *Oecophylla smaragdina*, rescue behaviour is mostly directed toward nestmates, but can also extend to conspecific non-nestmates from neighbouring colonies (Uy et al., 2019). In *C. nigra*, trapped pupae elicit stronger rescue responses than trapped adults (Bar et al., 2023). Furthermore, whereas lightly injured ants are rescued, severely injured ants are often not (Frank et al., 2018; Turza and Miler, 2023a; but see Bar et al., 2023, where both are similarly rescued). Not all individuals in a colony participate in rescue: some remain unresponsive even in the presence of a trapped nestmate, hinting at possible task specialization or behavioural variation in responsiveness among workers in *C. piliscapa* and *C. nigra* (Bar et al., 2023; Nowbahari et al., 2022). In *Megaponera analis*, dimethyl disulfide and dimethyl trisulfide stored in the mandibular gland reservoirs trigger rescue behaviour (Frank et al., 2017), suggesting that workers may vary in their response thresholds to pheromone concentration. In *Formica cinerea*, smaller workers exhibit greater persistence in rescue behaviour than larger individuals (Turza and Miler, 2023b), but the pheromones eliciting this behaviour do not seem to originate from the mandibular glands (Miler and Kuszewska, 2017). Rescue behaviour in ants such as *C. piliscapa* is consistent at the colony level, appears early in life and does not change with age (Andras et al., 2020). Furthermore, rescue behaviour in this species is heritable, and is associated with patrilineal origin (Andras et al., 2020), suggesting it is not simply a function of experience or development. A comparison of 14 European ant species from a broad phylogenetic background demonstrated that workers with longer life expectancy engage in rescue more frequently (Miler et al., 2017b; Turza et al., 2025), suggesting that some ant species exhibit a stronger specialization than others.

Here, we aimed to identify morphological, physiological and molecular factors that contribute to the expression of rescue behaviour in *Cataglyphis nigra* ant workers. We compared brain gene expression between individuals that engaged in rescue behaviour and those that did not respond to a restrained nestmate. Given the spatial and structural heterogeneity of the brain (Wang et al., 2024), with different brain regions having distinct neurological functions (Fischer et al., 2021), we focused on different brain tissues. We expected to find genes associated with decision making in the mushroom bodies, given their central role in multisensory integration and sensory-motor processing in insects (Arican et al., 2023; Hayashi et al., 2022). We also sequenced mRNA from the optic lobes, anticipating differential expression of genes related to sensitivity to visual cues from endangered nestmates and visual information processing (Wang et al., 2024), as *Cataglyphis* ants are known to rely on vision for orientation. Finally, we analysed the remaining central brain tissue (including the antennal lobes and central complex) given its role in modulating locomotion and processing of odorant information (Jaimes-Nino et al., 2024; Stone et al., 2017; Webb and Wystrach, 2016), and the antennae, because of their role in nestmate recognition and task specialization through the expression of odorant binding proteins and odorant receptors (Caminer et al., 2023; Stoldt et al., 2023). We found that differential gene expression in the mushroom bodies and optic lobes, and across all tissues may underlie the propensity to engage in rescue behaviour towards a restrained nestmate.

## MATERIALS AND METHODS

### Ethical statement

Animal care was in accordance with institutional guidelines. The experiments followed the rules of the animal protection law and official approvals were not necessary (no CITES species).

### Behavioural experiment

Seven queenright colonies of *Cataglyphis nigra* (André 1881; also referred to in some previous literature as *C. niger*) were collected from the Tel Baruch sand dunes (32.1283N, 34.7867E) in the winter of 2023. In these ant colonies, generally no brood is present during the winter season. Colonies were transferred to the laboratory and kept at ∼24°C, exposed to natural light in Plexiglas cages (50×20×10 cm). At least 1 week after collection, 40 worker ants of *C. nigra* (including a balanced number of individuals in the foraging area and inside the nest, but without queen or brood) per colony were separated and placed in an acclimation box (26×18×5 cm). Workers were randomly selected across a range of body sizes, and a coloured, numbered paper sheet (five colours, numbered 0–9) was glued to their thorax. Before the experiment started, the ants were given only water for 7 days during the acclimation to increase foraging activity. They were maintained at 28°C and 32% humidity, on a 12 h:12 h light:dark cycle. The colonies were tested between 9 and 30 November 2023. The acclimation box was set in front of the experimental arena, and cotton wool sealed the entrance. The experimental arena (15×20 cm) was covered with sand collected from Tel Baruch dunes. A worker from the original colony was tied 10 cm from the entrance, in the centre of the experimental arena. The cotton wool was removed, and the experiment was recorded from the time the first worker entered the arena for 15 min. Each ant that bit and/or pulled the tied individual (as in Bar et al., 2023; Miler et al., 2017a; Nowbahari et al., 2009) for 20 s was collected into a tube, shock-frozen in liquid nitrogen and stored at −80°C. After 15 min, all other ants that entered the arena were collected in tubes and similarly frozen and stored. Each run was recorded and tracked using the open software AnimalTA (v.3.2.2, Chiara and Kim, 2023) at 3.50 frames s^−1^.

**Fig. 1.**
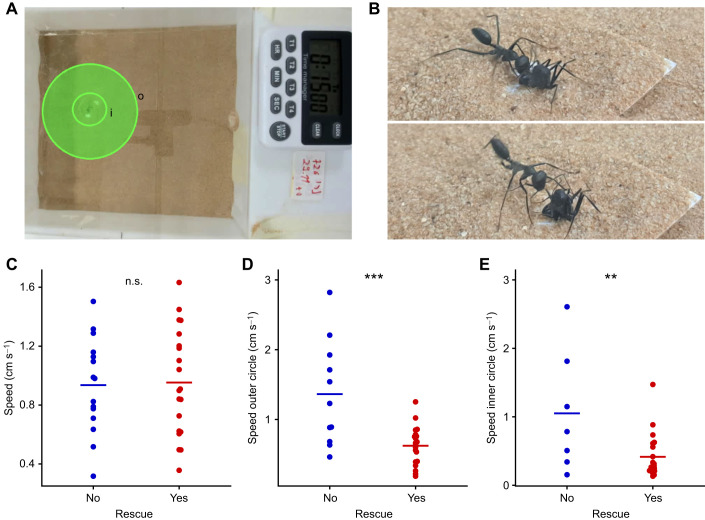
**Rescue behaviour in *Cataglyphis nigra* ants towards a restrained nestmate.** (A) Experimental setup with outer (diameter 10 cm) and inner circles (diameter 3 cm), the latter corresponding to half the length of the 10 cm arena. (B) A rescuer biting the thread around the tied individual (upper panel); a rescuer demonstrating pulling leg behaviour towards the endangered nestmate (lower panel). (C) Ant workers did not vary in their average speed (*N*=20 rescuers, *N*=15 non-rescuers, glmmTMB, χ^2^_1_=0.02, *P*=0.88). (D,E) Rescuers were slower than non-rescuers when moving within the outer circle ((D; *N*=20 rescuers, *N*=11 non-rescuers, χ^2^_1_=12.56, ***P<0.001) and the inner circle where the nestmate was located (E; *N*=20 rescuers, *N*=7 non-rescuers, χ^2^_1_=8.78, **P<0.01). Bars in C–E indicate means.

Rescuer and non-rescuer individuals were selected by delimiting two circle areas: an inner circle at a radius of ca. 1–1.5 cm from the tied individual and an outer one at 5 cm (Fig. 1A). A ‘rescuer’ was considered a worker that entered the inner circle and bit and pulled the tied individual and/or dug around the tied individual for at least 5 s. We compared them with ‘non-rescuers’, workers of the same colony that entered the outer circle, and therefore were close enough to perceive the endangered nestmate or its signals, but did not exhibit any pulling, biting and/or digging behaviour directed towards the tied nestmate. We extracted the average speed through the whole run, and when traversing the circles using the software AnimalTA. The workers' path was smoothed using the Savitzky–Golay filter in the program.

We tested for differences among rescuers and non-rescuers in general speed, speed when traversing the inner and outer circle (log transformed), the proportion of the arena explored, the proportion of the inner and outer circle explored, the total travelled distance (log transformed), and the latency to reach the outer and inner circle using linear models with Gaussian distribution (link=identity), including the video ID as a random variable, with the function glmmTMB [glmmTMB package v.1.1.10 (Brooks et al., 2017) in R]. No transformation was applied to the response variables unless otherwise specified. In all cases, model assumptions were visually inspected using the function simulateResiduals and plotting the QQ residuals (DHARMA package v.0.4.6; https://CRAN.R-project.org/package=DHARMa), checking for adequate distribution (KS test), dispersion and outliers' test. We compared the full model containing the predictor ‘rescue’ with simpler models without the term using the anova function (R stats package v.4.5.1). We examined how movement patterns differed between workers that exhibited rescue behaviour and those which did not, to later identify genes whose expression was associated with the likelihood of showing rescue behaviour.

### Sensitivity analysis

We performed a sensitivity analysis to determine whether the average speed estimation showed similar patterns between rescuers and non-rescuers when analysing the videos at a higher resolution. For this, we randomly selected four out of the seven videos, and we tracked the movement of six rescuers and six non-rescuers. We analysed the movement at the maximum frame resolution possible (28 frames s^−1^) and compared the speed at 3.5 frames s^−1^. We used mixed linear models with rescuer/non-rescuer, the resolution (lower or higher) and the interaction of rescue behaviour and resolution as explanatory fixed variables, and the combination of colony ID and individual ID as a random variable. To test whether the resolution altered the rescuer versus non-rescuer comparison of average speed (log transformed), and speed when traversing the inner (log-transformed) and the outer circle (log-transformed), we used linear models with Gaussian distribution (identity) using the function glmmTMB as previously described. We compared the full model (two predictors and the interaction) with simpler sub-models without the evaluated terms using the anova function (R stats package v.4.5.1). Model assumptions were checked as described above.

### RNA extraction and sequencing

We pooled tissue from two workers of the same colony that were selected in the behavioural category to obtain seven samples of rescuers and non-rescuers. Therefore, brains and antennae of 28 workers were selected for RNA extraction. First, the antennae were cut off and stored in 50 μl TRIzol (Invitrogen) at −80°C. An incision was made on the head cuticle at the antennal base up to the ocelli and compound eyes. The cuticles, glands and muscle tissue around the brain were removed, and the exposed tissues of the head were submerged in Dulbecco's Phosphate-Buffered Saline (DPBS; 1×, Gibco). The brain was photographed to measure its width and length. Then, the optic lobes (OL) were removed and stored in 25 μl TRIzol. The peritoneal sheath, enclosing the brain and mushroom bodies, was next pinched to release the mushroom body calyces and peduncles (MB). The MB corresponded mainly to the calyces, where a large part of the mushroom body transcription takes place, given the difficulty in extracting the complete peduncles. The remaining central brain tissue, including antennal lobes and central complex (hereafter collectively referred to as the CC), was placed in 25 μl of TRIzol. We did not dissect the antennal lobes separately, although this tissue receives olfactory input and may underlie differential responses to endangered individuals, because it is difficult to consistently separate it from the central brain. Each dissection was completed in less than 15 min to prevent RNA degradation.

The antennae were homogenized manually with plastic pistils, and the brain tissues were homogenized using a lyophilizer at 20 oscillations s^−1^ for 1 min. Total RNA was extracted using the Directzol RNA miniprep Zymo following standard instructions. Afterward, samples were sent to Novogene (Planegg, Germany) for RNA quality control, library construction (poly A enrichment), and sequencing at 150 bp paired-end reads on an Illumina NovaSeq6000. We obtained a total mRNA yield of 57 ng on average from the OL, 51 ng from the MB, 88 ng from the CC and 45 ng from the antennae. Two samples were excluded from sequencing because of a mismatch during pooling (OL_D_727 and CC_E_718), and one sample failed during library preparation (MB_B_724). Reads were adapter and quality trimmed using TrimGalore (v.0.6.7), and an average of 37 million reads per sample was obtained. Read quality was assessed using FastQC (v.0.12.1) and summarized using MultiQC (v.1.11.). Trimmed reads were mapped against the reference genome of *C. nigra* (v.3.1; Cohen et al., 2025) using STAR (v.2.7.11b; Dobin et al., 2013) with the genome-mode, using default parameters and specifying --quantMode GeneCounts, for later use in DESeq2 analysis. We mapped the counts to 14,889 genes and obtained an average of 91.3% of back mapping for the OL, 91.7% for the MB, 89.9% for the CC and 87.6% for the antennae. We filtered out genes with counts below 10 reads in at least six samples across all samples and tissues, leaving a gene set of 10,713 genes in all tissues for further DESeq2 analysis.

### Analysis of gene expression

Gene expression associated with rescuer/non-rescuer behaviour was tested using the likelihood ratio test (LRT) with the package DESeq2 v.1.46.0 (Love et al., 2014). The influence of colony identity was controlled for by including it as an explanatory variable in the full and reduced models. We assessed genes whose expression variation was significantly better explained by a full model incorporating our fixed effects (∼Rescue+Colony ID) compared with a reduced model (∼Colony ID). Expression differences were considered significant at a false discovery rate (FDR)-adjusted *P*-value of <0.05 using the Benjamini–Hochberg method to adjust *P*-values for multiple testing (Benjamini and Hochberg, 1995). Principal component analysis (PCA) plots were produced to visualize the samples after variance stabilizing transformation. To test for differences in gene expression associated with rescuer behaviour in individual tissues, we filtered out genes with counts below 10 in at least six samples within the tissue, independent from the previous filtering, for further DESeq analysis. Further, we updated the *C. nigra*  annotation (v.3.1) (Cohen et al., 2025) using Interproscan to obtain Gene Ontology (GO) terms, protein families and domain annotations. Additionally, we used BLAST (NCBI) on the nucleotide sequence for the longest non-annotated differentially expressed gene (DEG) transcript and reported the best hit based on the lowest E-score, coverage and sequence identity. Lastly, we performed a GO enrichment analysis, including the newly predicted GO terms. All analyses were performed in R v.4.4.3 (https://www.r-project.org/).

### Morphological data

Ten additional colonies of *C. nigra* were collected from Tel Baruch sand dunes in the winter of 2025 and brought to the laboratory. Similar to the behavioural experiment, a group of 40 workers of random size was separated from each colony (without brood and queen) and placed into an acclimation box. All the workers in the experiment were marked with coloured paper glued on the thorax. The acclimation and the experiment were done according to the same protocol as the behavioural experiment. At the end of each group trial, two rescuers and two non-rescuers were collected and stored at −20°C for subsequent morphological analysis. In total, 19 rescuing workers and 12 non-rescuers were collected, and the following traits were measured: right hindtibia length (mm), thorax length (mm), head width (mm), mandible length (mm), dry mass (g) and lipid proportion. To obtain the worker's dry mass, we first dried the workers at 60°C for 2 days and then weighed each using a Precisa scale (to the nearest 0.0001 g). Then, we placed each worker in 1 ml of petroleum ether for 5 days to extract non-polar lipids using the gravimetric method (Williams et al., 2011), dried the worker again at 60°C, and weighed it to obtain the lipid-free mass. The difference in mass provides an estimate of mass of the non-polar lipids. We used PCA to summarize variation in morphological traits. The PCA resulted in two principal components (PCs) with an eigenvalue larger than 1 (Table S1). We then used two linear mixed models with rescuer/non-rescuer as the explanatory fixed variable, colony ID as the random variable, and either PC1 or PC2 as the response variable.

## RESULTS

### Behavioural and morphological differences

Workers were classified as rescuers if they entered the 1–1.5 cm inner circle and bit and pulled the tied individual, and/or dug around the restrained nestmate for at least 5 s (Fig. 1B). Non-rescuers were individuals that entered the 5 cm outer circle without displaying rescue behaviour during the whole run (Fig. 1A). Rescuer and non-rescuer workers did not differ in their average speed when exploring the arena (glmmTMB, χ^2^_1_=0.02, *P*=0.88; Fig. 1C) but differed in the speed when traversing the outer circle (χ^2^_1_=12.56, *P*<0.001; Fig. 1D) and the inner circle where the endangered nestmate was tied (χ^2^_1_=8.78, *P*<0.01; Fig. 1E). Rescuers were on average 0.50 cm s^−1^ slower within the outer circle compared with non-rescuers.

**Fig. 2.**
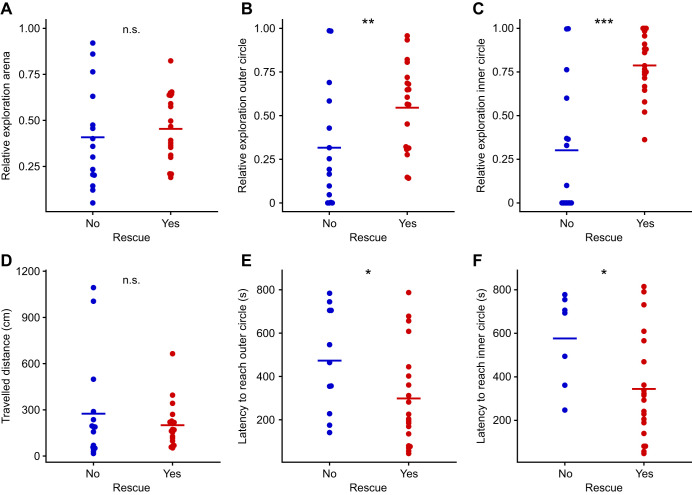
**Differences between rescuers and non-rescuers in arena exploration, travelled distance and latency to reach the restrained nestmate.** Data were analysed using AnimalTA. (A–C) Workers did not differ in the proportion of the arena explored (A; glmmTMB, χ^2^_1_=0.22, *P*=0.64) but rescuers explored a larger proportion of the outer (B; χ^2^_1_=6.87, **P<0.01) and inner circle (C; χ^2^_1_=21.37, ***P<0.001; *N*=20 rescuers, *N*=15 non-rescuers). (D–F) Rescuers and non-rescuers did not differ in total travelled distance (D; *N*=20 rescuers, *N*=15 non-rescuers, glmmTMB, χ^2^_1_=0.13, *P*=0.72) but rescuers showed lower latency to approach the outer (E; *N*=20 rescuers, *N*=11 non-rescuers, glmmTMB, χ^2^_1_=5.95, *P<0.05) and inner circle (F; *N*=20 rescuers, *N*=7 non-rescuers, χ^2^_1_=6.58, *P<0.05) where the nestmate was located.

Rescuers and non-rescuers did not differ in the proportion of the arena explored (glmmTMB, χ^2^_1_=0.22, *P*=0.64), but rescuers explored 24% more area of the outer circle (glmmTMB, χ^2^_1_=6.87, *P*<0.01) and 49% more area of the inner circle (glmmTMB, χ^2^_1_=21.37, *P*<0.001), compared with non-rescuers (Fig. 2A–C). Additionally, while their total travelled distance did not differ (glmmTMB, χ^2^_1_=0.13, *P*=0.72; Fig. 2D), rescuers reached the outer circle 3 min (s.e.m. ±72 s), and the inner circle 4 min (s.e.m. ±90 s) faster than non-rescuers (glmmTMB, outer circle χ^2^_1_=5.95, *P*<0.05; inner circle χ^2^_1_=6.58, *P*<0.05; Fig. 2E,F).

A sensitivity analysis of six rescuers and six non-rescuers tracked at 28 frames s^−1^ yielded similar results, with rescuers traversing both the outer and inner circle slowing down in the area close to the entrapped nestmate (glmmTMB; rescuer type, outer circle: χ^2^_1_=11.34, *P*<0.001; inner circle: χ^2^_1_=6.20, *P*<0.05). Videos tracked at a higher frame rate produced higher estimates of ant speed throughout the run (estimated marginal means±s.e.: higher frame rate 1.20±0.09 cm s^−1^; lower frame rate 0.88±0.06 cm s^−1^), probably because the higher temporal resolution captured more detailed movements (video resolution effect on general speed, χ^2^_1_=14.99, *P*<0.001). This effect was also observed when ants traversed the outer circle (video resolution, χ^2^_1_=4.34, *P*<0.05) and inner circle (χ^2^_1_=6.51, *P*<0.05). However, the reduction in speed in rescuers relative to non-rescuers was independent of video resolution (resolution×rescuer type, general speed: χ^2^_1_=1.50, *P*=0.22; outer circle: χ^2^_1_=1.57, *P*=0.21; inner circle: χ^2^_1_=0.90, *P*=0.34; Fig. S1).

Brain dissections and morphometric measurements revealed that rescuers and non-rescuers did not differ in brain size (brain length glmmTMB, χ^2^_1_=0.29, *P*=0.59; Fig. S2A,B). We used a PCA to summarize variation in morphological (head width, tibia, thorax and mandible length; Fig. S5A,B) and physiological traits (body mass and lipid proportion). We then used linear mixed models to test for differences between rescuers and non-rescuers in PC1 and PC2. While neither axis exhibited a significant association with rescue behaviour, PC1 exhibited a non-significant trend, in that larger workers were slightly more likely to exhibit rescue behaviour (PC1 *t*=0.080, *P*=0.094, PC2 *t*=−0.543, *P*=0.600). The first axis represented overall body size, whereas the second axis reflected body reserves, primarily driven by the thorax length and lipid content (Table S1 and Fig. S3).

### Gene expression differences across tissues

**Fig. 3.**
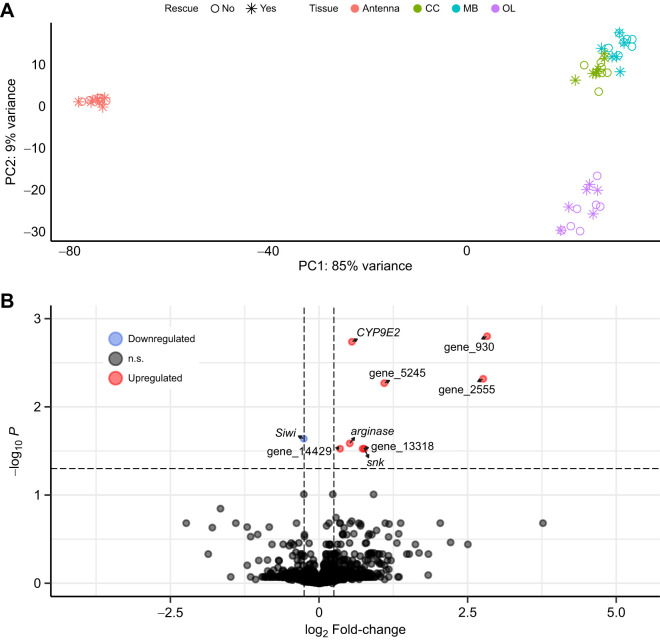
**Principal component analysis (PCA) plot and differentially expressed genes (DEGs) from the brain and antennae.** (A) Variance-stabilized transformed genes expressed in pooled *C. nigra* ants (two workers) in the optic lobes (OL), mushroom bodies (MB), central brain including the central complex (CC) and the antennae, and their rescue behaviour. PCA based on the top 500 genes with larger variance across all expressed genes. (B) Volcano plot of gene expression across all tissues. Positive log_2_ fold-change values (red) correspond to genes that were upregulated, while negative values (blue) correspond to downregulated genes in rescuers when compared with non-rescuers (vertical dashed line at 0.25 and −0.25 threshold). Significant differences were calculated using the likelihood ratio test (LRT) and corrected for multiple testing using the Benjamini–Hochberg method (i.e. adjusted *P*<0.05, horizontal dashed line). *snk*, serine protease snake gene; *Siwi*, piwi-like protein Siwi gene; *CYP9E2*, cytochrome P450 9e2 gene.

PCA of the combined tissues revealed clear separation of expression profiles among the different tissues (PC1 and PC2 both with λ>1; Fig. 3). PC1 explained 85% of expression variance, indicating strong differences between the brain parts (OL, MB and CC) and the antennae. PC2 explained only 9% of variance, indicating less variation captured by the analysis and a stronger divergence between OL and both MB and CC.

We compared full models that included rescue with reduced models without it, using the LRT method implemented in DESeq2. The tissue identity better characterized 9913 DEGs out of a total of 10,713 expressed genes. Additionally, we found nine DEGs in the combined analysis whose expression was explained by rescue behaviour (Fig. 3B; Table S2). Among those nine genes, six were not differentially expressed in tissue-specific analyses. The genes encoding serine protease snake (*snk*, gene_1997), cytochrome P450 9e2 (*CYP9E2*, gene_14458), arginase (gene_7603), and the non-annotated genes gene_13318 and gene_14429 were upregulated, and that encoding the piwi-like protein Siwi (*siwi*, gene_13632) was downregulated in rescuers compared with non-rescuers when comparing all tissues together.

### Gene expression differences per tissue

**Fig. 4.**
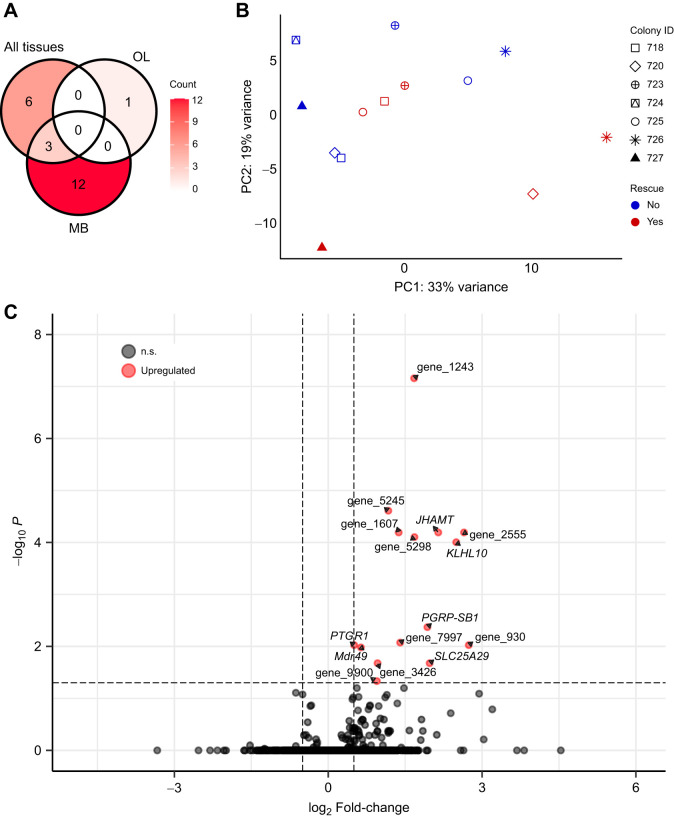
**Gene expression patterns in rescue and non-rescue workers across tissues and in the MB.** (A) Venn diagram of DEGs associated with rescue behaviour across all tissues, the MB and the OL. (B) PCA plot based on the top 500 variance-stabilized transformed genes expressed in the MB. (C) Volcano plot of gene expression in the MB. Positive log_2_ fold-change values (red) correspond to genes upregulated in rescuers when compared with non-rescuers (vertical dashed line at 0.5 and −0.5 threshold). Significant differences were calculated using the LRT and corrected for multiple testing using the Benjamini–Hochberg method (i.e. adjusted *P*<0.05, horizontal dashed line). *KLHL10*, Kelch-like protein 10; *SLC25A29*, mitochondrial basic amino acid transporter; *DEFA*, defensin; *JHAMT*, juvenile hormone acid *O*-methyltransferase; *Mdr49*, multidrug resistance protein homolog 49; *PTGR1*, prostaglandin reductase 1; *PGRP-SB1*, peptidoglycan-recognition protein SB1.

To examine tissue-specific expression differences, we conducted a DESeq2 analysis for each tissue separately. The LRT revealed significant differential expression in the MB and OL, but not in the CC or antennae (see Tables 7–10 in Dataset 1). In the MB, 15 genes were differentially upregulated in rescue workers, while in the OL, one gene showed differential upregulation. Of these, three genes were consistently differentially expressed in both the MB and the combined tissue analysis (Fig. 4A): defensin gene (*DEFA*, gene_930), endocuticle structural glycoprotein SgAbd-1 gene (*SgAbd-1*, gene_5245) and the non-annotated gene_2555. In the MB, the first principal component (PC1) explained 33% of the variance in gene expression, while the second component (PC2) accounted for an additional 19% (both axes with λ>1; Fig. 4B).

The 12 non-shared upregulated genes in the MB corresponded to those encoding Kelch-like protein 10 (*KLHL10*, gene_3661), mitochondrial basic amino acid transporter (*SLC25A29*, gene_3662), multidrug resistance protein homolog 49 (*Mdr49*, gene_6089), prostaglandin reductase 1 (*PTGR1*, gene_13149), juvenile hormone acid *O*-methyltransferase (*JHAMT*, gene_9895) and peptidoglycan-recognition protein SB1 (*PGRP-SB1*, gene_8499). The unannotated genes differentially upregulated in the MB were gene_3426, gene_1607, gene_1243, gene_5298, gene_9900 and gene_7997 (Fig. 4C). In terms of colony identity, a full model with the fixed terms ‘colony identity’ and ‘rescue behaviour’ better fitted the expression profiles of 463 DEGs in the MB, compared with a model without ‘colony identity’. The unique upregulated gene in the OL corresponded to the non-annotated gene_5301.

### Non-annotated DEGs and GO-term enrichment

We used the predicted protein sequence of the ten non-annotated DEGs to gain insight into predicted protein families, domains and/or functional sites. For seven of these genes, we found annotations corresponding to InterPro entries (IPR), Pfam protein families, PANTHER protein models and/or GO terms. Specifically, we predicted the Interpro query Pheromone/general odorant binding protein (IPR006170) and odorant binding GO term (GO:0005549) for three of the genes upon scanning against the Interproscan protein signature databases (Table S2). Two of the genes coding for odorant binding proteins were upregulated in the MB, and one in the OL. Other predicted family members found were the Tumor Necrosis Factor Ligand Superfamily for gene_9900, and Prokaryotic membrane lipoprotein lipid attachment site profile (PS5125) in gene_1243.

To gain deeper insight into the biological and molecular processes of the genes upregulated in rescuers, we performed a GO enrichment analysis, including the newly predicted GO terms. The upregulated genes in the MB were significantly enriched (*P*<0.05) for biological functions related to immune response (3 of 28 annotated genes), social behaviour and the peptidoglycan catabolic process. Additional enriched processes included prostaglandin metabolism, transmembrane transport of mitochondrial l-ornithine, and oligopeptide export from mitochondria. Enriched molecular functions involved 2-alkenal reductase [NAD(P)+], 15-oxoprostaglandin 13-oxidase [NAD(P)+] and *N*-acetylmuramoyl-l-alanine amidase activities, as well as binding to peptidoglycan and the tumour necrosis factor receptor. The associated cellular components were primarily the extracellular region and the chitin-based extracellular matrix (Table S2).

## DISCUSSION

Division of labour is a key organizational principle in insect societies, typically associated with variation in body size, age, experience and molecular physiology among workers (Robinson and Jandt, 2021; Seid et al., 2005; Wilson, 1976). However, workers may also vary in their propensity to perform rare and risky tasks. Here, we investigated which traits distinguish individuals that engage in rescue behaviour from their non-helping nestmates. Behaviourally, rescuers differed from non-rescuers when approaching a restrained nestmate. They slowed down and explored more locally yet showed no differences in overall exploration or average speed across the arena. Despite the limited sample size, this pattern even held when the videos were analysed at higher temporal resolution. Importantly, rescuers reached the trapped individual faster, suggesting increased sensitivity or responsiveness to visual, auditory or odour cues emitted by the nestmate.

In contrast, we found no evidence that rescue behaviour is associated with morphological or energetic differences, as body and brain size, body mass and lipid content did not significantly differ between groups. We observed a non-significant trend that rescuers were of larger body size. A larger sample size and more refined morphometric measurements – for example, including specific brain parts associated with odorant perception – might reveal variation in which body parts are linked to rescue behaviour.

However, we detected differential expression of a few genes across tissues, with several specific to the MB, one to the OL and no genes exclusive to the antennae or CC. Our results suggest that genes involved in polyamine synthesis, immune regulation and transposon activity appear to contribute to information processing and subsequent decision making critical for underlying rescue behaviour.

### DEGs involved in polyamine synthesis

We elucidated an upregulation of arginase in the combined tissues and of the mitochondrial basic amino acid transporter in the MB of the rescuers, as well as an enrichment of high-affinity lysine and the mitochondrial l-ornithine transmembrane transporter. Arginase increases ornithine availability, a precursor for polyamines and proline. Polyamines modulate the phosphorylation of antennal proteins, the sensitivity to female pheromones (Zhang et al., 2015, 2008) and memory retrieval (Pratavieira et al., 2020). In general, the molecular role of polyamines seems diverse (Miller-Fleming et al., 2015), as they can interact with nucleic acids and proteins, possibly playing an important role in the regulation of gene expression and odour processing.

### DEGs with known immune-related functions

We uncovered upregulation of several immune-related genes in the MB of rescuers and in the combined tissue analysis. These included genes encoding proteins involved in prokaryote recognition and immune responses, such as defensin (*DEFA*), peptidoglycan-recognition protein SB1 (*PGRP-SB1*) and a previously unannotated gene (gene_1243) encoding a prokaryotic membrane lipoprotein attachment site profile. Additional genes, such as those encoding the multidrug resistance protein homolog 49 (*Mdr49*), a P-glycoprotein efflux transporter, and the serine protease snake (*snk*), contribute to the maintenance of the blood–brain barrier in insects (Al-Qadi et al., 2015; Andersson et al., 2014; DeSalvo et al., 2014; Guseman et al., 2016; Kanost and Jiang, 2015; Wang et al., 2018).

Immune signalling can directly influence neural function and behaviour. In insects, bacterial peptidoglycans modulate the central nervous system via the gut–immune–brain axis (Fioriti et al., 2024), while in vertebrates similar pathways are linked to behavioural and neurodevelopmental changes (Hsiao et al., 2013; Sharon et al., 2019; Tartaglione et al., 2022). Here, genes associated with anti-inflammatory and tissue-protective metabolic responses, including *PGRP-SB1*, prostaglandin reductase 1 (*PTGR1*), Kelch-like protein 10 (*KLHL10*) and the newly annotated gene_9900 were also upregulated (Royet and Dziarski, 2007; Vitturi et al., 2013). In flies, PGRPs and Eiger, a homolog of TNF-α (*eiger* gene_9900) are required for homeostatic synaptic plasticity, enabling neurons to regulate their excitability (Harris et al., 2015; Stellwagen and Malenka, 2006), while *eiger* expression also promotes glial proliferation during development (Keller et al., 2011). More broadly, immune gene networks have been associated with brain development and social behaviour in mice, influencing traits such as preference for social novelty (Ma et al., 2015).

Together, these findings suggest that enhanced immune signalling in rescuers may support neural homeostasis and plasticity, particularly within the MB, thereby modulating responsiveness to social stimuli.

### Downregulation of transposon silencing in rescuers

We observed a downregulation of *siwi*, encoding the Piwi-like protein SiWi (silkworm Piwi), an ortholog of the Aubergine gene (*aub*) in *Drosophila*. Piwi-family Argonaute proteins are involved in post-transcriptional silencing of transposons (Matsumoto et al., 2016; Yamashiro and Siomi, 2018). Consistent with this, higher transposon expression in the αβ neurons of the MB has been suggested to result from a lower abundance of Aubergine compared with adjacent neuronal regions (Perrat et al., 2013). Although transposon activity has recently been linked to neurological disorders (Erwin et al., 2014; Sun et al., 2018; Treiber and Waddell, 2020), its behavioural consequences remain unclear. Our results raise the possibility that variation in transposon regulation contributes to neuronal heterogeneity and behaviour differences.

### Genes with non-canonical functions

Several DEGs identified here have functions not typically associated with the tissues in which they were expressed, suggesting potential non-canonical roles. Cytochrome P450 9e2 (*CYP9E2*) maintains the sensitivity and accuracy of the olfactory system by degrading and detoxifying exogenous compounds (Huber et al., 2007; Kang et al., 2021; Kuang et al., 2022; López et al., 2013; Maïbèche-Coisne et al., 2004). Although odorant-degrading enzymes are typically expressed in the antennal sensilla, no differential expression was detected in the antennae despite high baseline expression (Fig. S4). This discrepancy may reflect limited statistical power or suggest a previously uncharacterized function of *CYP9E2* in central processing.

Similarly, previously unannotated genes upregulated in the MB and OL of rescuers may encode odorant-binding proteins. Although originally identified in the olfactory sensilla (Vogt and Riddiford, 1981), these proteins are also expressed in other tissues, including gustatory organs, legs, glia and the peripheral sensory system, where they may influence neuronal signalling and lipid metabolism (Koganezawa and Shimada, 2002; Matsuo et al., 2007; Park et al., 2024). Their presence in higher-order brain regions suggests a role in sensory integration rather than primary detection, although these functions remain to be experimentally validated.

We also found upregulation of juvenile hormone acid *O*-methyltransferase (*JHAMT*) in the MB of rescuers. *JHAMT* produces active juvenile hormone (JH) in the corpora allata of insects (Niwa et al., 2008; Zhang et al., 2022), which regulates metamorphosis, behaviour (Guo et al., 2020; Zhang et al., 2022) and pheromone perception (Gadenne et al., 1993). The application of methoprene, a JH homolog, influences MB expansion in honeybees and flies (Withers et al., 1995; Wu et al., 2021). These results point to a role for JHAMT in the neuropils that was previously undescribed. Finally, the structural glycoprotein SgAb-1, upregulated across tissues, typically contributes to the endocuticle structure of insects. Our study is the first to suggest a role of SgAb-1 in insect behaviour, but its function in the MB remains unknown.

Our findings align with previous evidence that rescue behaviour can be specialized within ant colonies. In species of the genus *Cataglyphis*, rescue behaviour follows complex sequences and may be genetically inherited (Andras et al., 2020; Bar et al., 2023; Nowbahari et al., 2022, 2012). Task specialization may extend beyond common activities: in *C. nigra*, fewer workers than expected perform both foraging and rescuing, indicating division of labour at the colony level (Bar et al., 2023). Life-history traits also appear relevant, as species with longer-lived workers are more likely to rescue and be rescued (Turza et al., 2025), whereas individuals with shortened lifespans show reduced rescue behaviour (Miler et al., 2017a), suggesting increased social withdrawal. Such specialization may enhance colony efficiency by restricting high-risk tasks to particularly responsive individuals.

Rescue behaviour is commonly triggered by vibrational and/or chemical cues. The differential expression of genes involved with odour processing and neuromodulation, particularly within the MB, supports the idea that rescuers differ in how they integrate and respond to these signals. These differences are unlikely to be a result of rescue, as transcription was halted immediately after the behaviour was observed. Likewise, they are unlikely to reflect differences in time spent in the arena, given that transcriptional responses generally require 20 min to several hours to manifest (Bahrami and Drabløs, 2016; O'Brien and Lis, 1993; Walton et al., 1999).

It remains unknown whether all nestmates would eventually exhibit rescue behaviour under longer exposure times (e.g. beyond 15 min). However, within the time frame examined here, clear behavioural and molecular differences were evident. Overall, rescue behaviour appears to be linked to variation in neural processing rather than morphology or general activity. Pathways related to polyamine metabolism, immune signalling and transposon regulation, together with gene expression in non-canonical tissues, point to complex modulation of sensory integration and decision making.

### Conclusion

We investigated rescue behaviour in ants, exhibited by some, but not all, individuals within 15 min of exposure to a distressed nestmate. These individual differences could not be explained by variation in morphology or lipid content but were instead associated with differences in the expression of sensory processing genes, among others. Our findings highlight the involvement of genes related to polyamine synthesis and humoral immunity in modulating the propensity to engage in rescue behaviour in ants. The upregulation of genes in non-canonical tissues, such as the juvenile hormone acid *O*-methyltransferase gene *JHAMT* and previously unannotated genes with odorant-binding functions in the MB, suggests novel molecular functions in the neuropils of rescuers, which require further corroboration. Nevertheless, we show that the expression of genes associated with odorant perception in non-canonical tissues is linked to the likelihood that an individual responds to an endangered nestmate.

Insects' humoral immune response genes are known to be involved not only in pathogen defence but also in modulating behaviour, including sleep, activity and social aggregation. Recent studies have emphasized the role of immune regulation of the gut microbiota in shaping social behaviour via the gut–brain axis. It is highly plausible that immune-related genes are co-adapted to modulate behaviour even in the absence of infection. An alternative explanation is that ants engaging in rescue behaviour may face an increased risk of injury or infection, making the upregulation of immune pathways potentially adaptive.

## Supplementary Material

10.1242/jexbio.252086_sup1 Supplementary information

Dataset 1. Raw data and results of “Transcriptional predictors of rescue behaviour in ants”.
