# Supplementary material for: Transcriptional predictors of rescue behaviour in ants
Source: J Exp Biol. 2026 Jul 17;229(14):jeb252086. doi: 10.1242/jeb.252086 (PMC13405234; doi:10.1242/jeb.252086)
Supplement: Supplementary information [file jexbio-229-252086-s1.pdf]

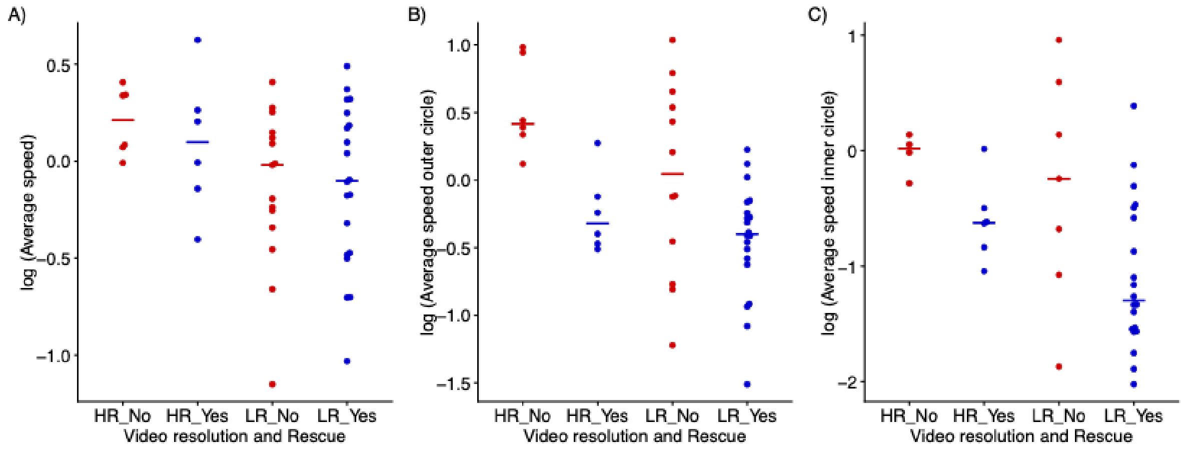


**Fig. S1. Average speed of rescuer and non-rescuer ants in videos tracked at different resolution frame (HR = 28 frames/second, LR = 3.5 frames/second,** $N_{HR}$ = 6, $N_{LR}$ =35**).** A) Average ant speed (log-transformed (cm/s)) between rescuers and non-rescuers tracked a high- and low-resolution frame rate in the whole run, B) traversing the outer circle, and C) traversing the inner circle. Crossbar indicates the median.

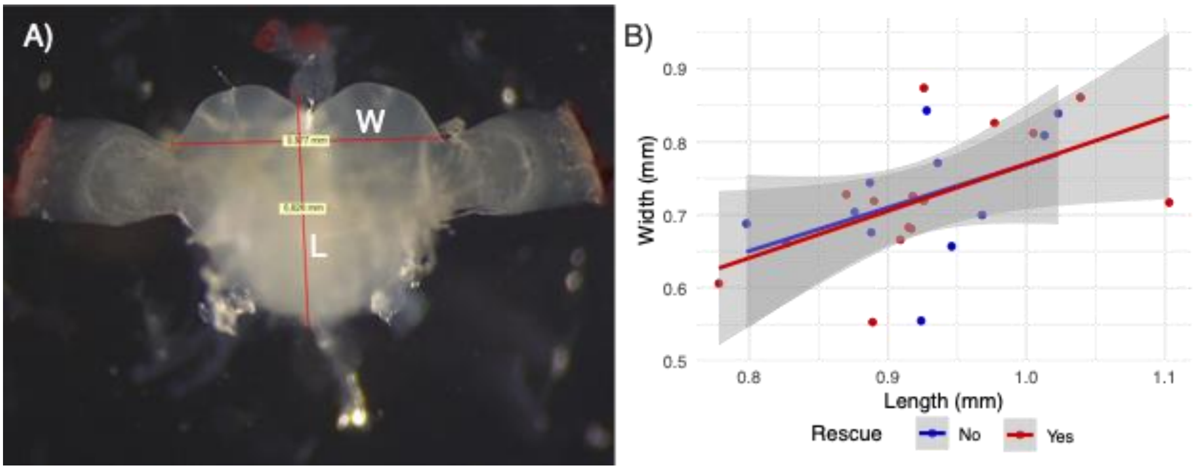


**Fig. S2. Brain width (W) and length (L).** A) Each dissected brain was photographed and measured during the dissection. B) No differences in brain size were found between brains of rescuers vs non-rescuers (lme, z-value = 0.55, p = 0.58). The band represents the 95% confidence interval.

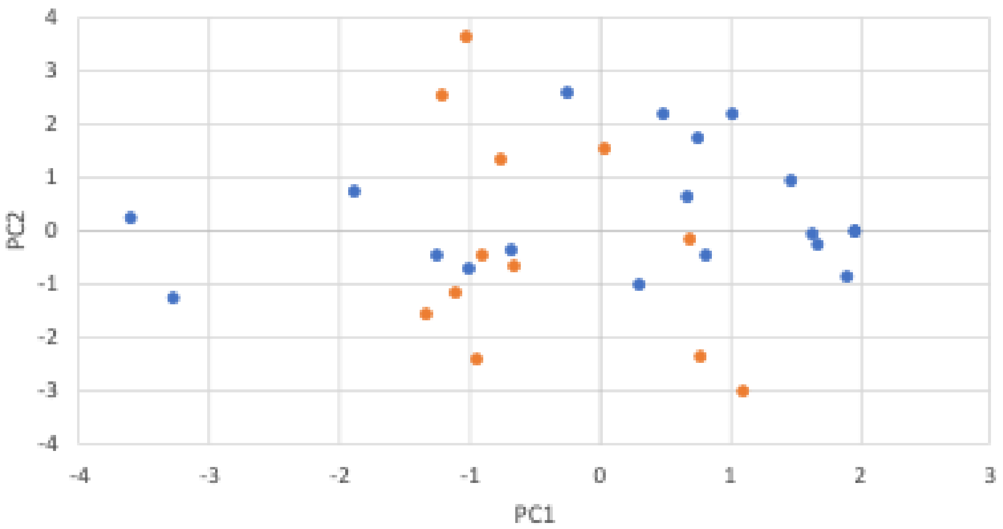


**Fig. S3. Principal component analysis plot of morphological and physiological variation between rescuers (blue) and non-rescuers (orange).** PC1 represents general body size (positive values representing larger body size) and describes 56,2% of the variance, whereas PC2 mostly represents lipid proportion and thorax length, and represents 19,6% of the variance. Neither PC1 (t = 0.080, p = 0.094) nor PC2 (t = −0.543, p = 0.600) were statistically significantly associated with rescuing behaviour.

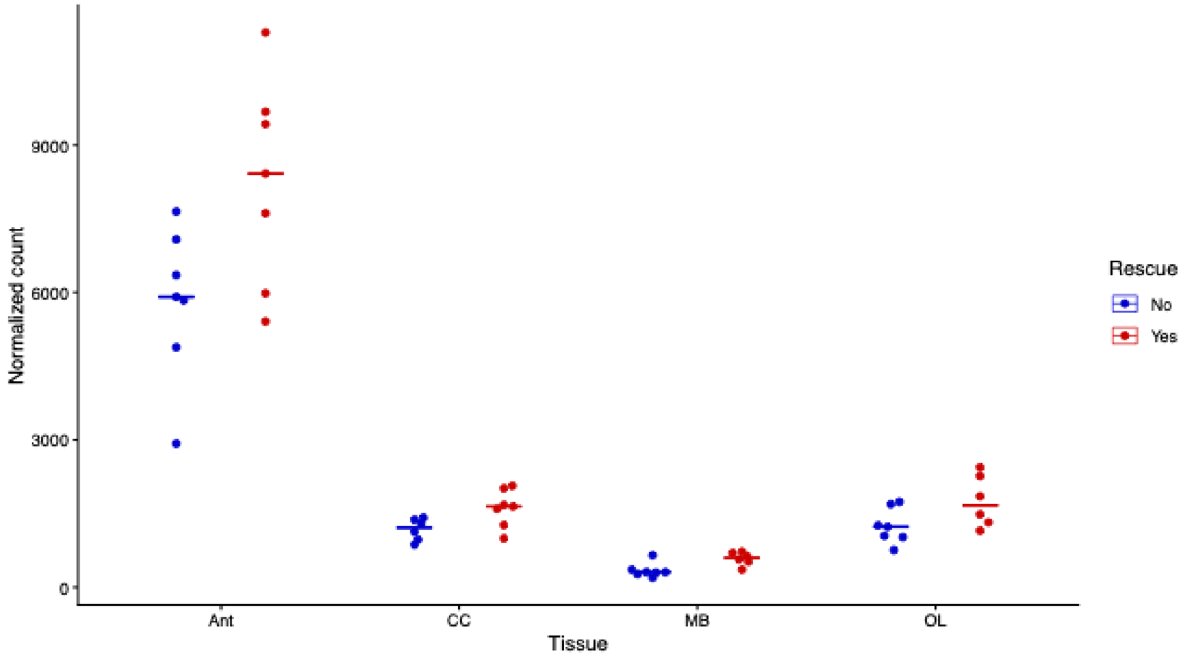


**Fig. S4.** ***Cytochrome P450 9e2*** **(gene 14458) expression is related to rescue behaviour across all tissues.** Normalized counts showed the antennae as the tissue with the highest expression of the gene *Cytochrome P450 9e2* (Supplementary File 1, Table 1), but significant expression differences among groups were only encountered in the combined tissue analysis (FDR, $p < 0.05$, Supplementary File 1, Tables 6-9). The bars describe the mean per tissue and per group.

Journal of Experimental Biology • Supplementary information

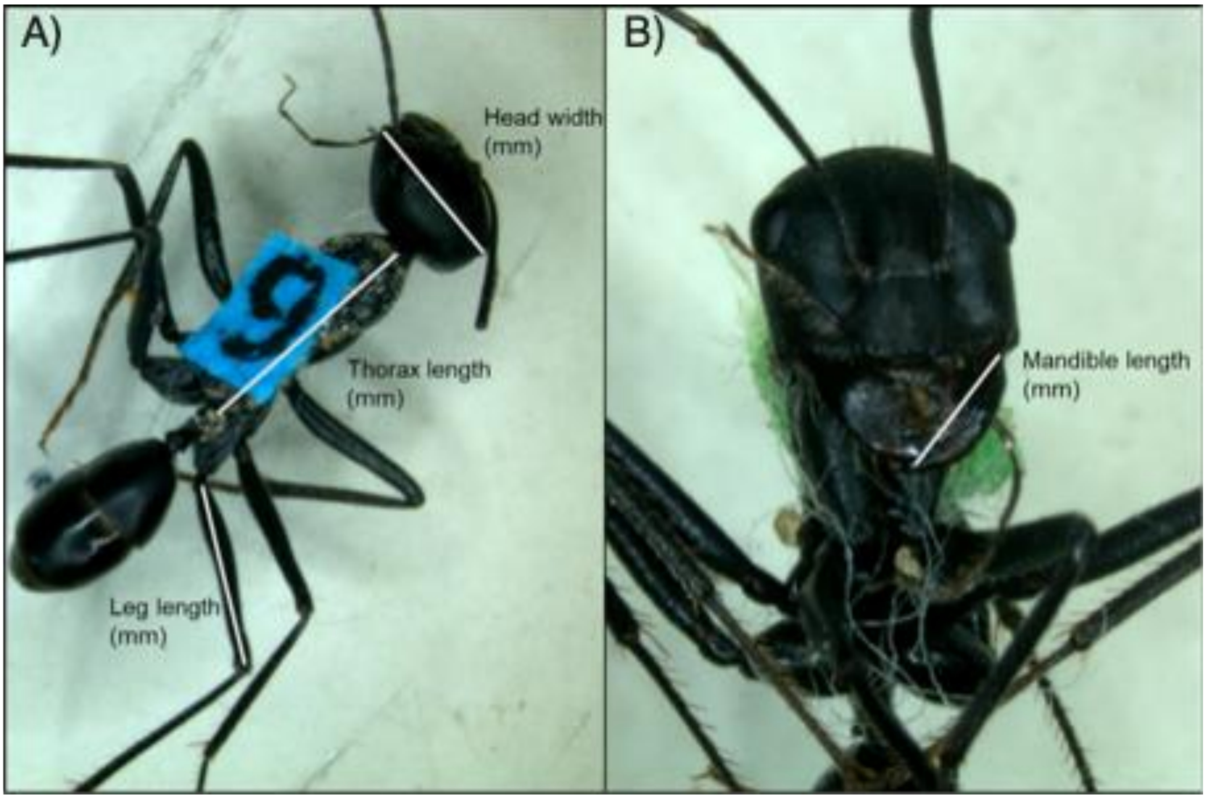


**Fig. S5. Morphological measurements of *Cataglyphis niger* ant workers.** A) The head width, thorax length, tibia length of the right hind leg, and B) mandible length for 31 workers were measured (rescuers = 19, and non-rescuers = 12 workers).

**Table S1.** Principal components of the principal component analysis (PCA) done on the morphological traits.

| | PC1 | PC2 |
|---|---|---|
| Eigenvalue | 3.3721 | 1.1785 |
| % variance explained | 56.2% | 19.6% |
| Tibia length | 0.4243 | -0.0610 |
| Thorax length | 0.3746 | 0.3758 |
| Head width | 0.4913 | -0.1477 |
| Mandible length | 0.4507 | -0.1851 |
| Body mass | 0.4740 | 0.2707 |
| Lipid proportion | -0.1024 | 0.8519 |

Journal of Experimental Biology • Supplementary information

**Table S2. Predicted protein family and domains from previously non-annotated upregulated genes predicting for recue-behaviour.** Blast best hit for the nucleotide longest sequence based on -value, sequence identity, query coverage, and alignment score.

| Genes | Best hit (BLAST) | Interproscan Pfam domains | GO terms |
|---|---|---|---|
| All tissues | | | |
| gene_2555 | No highly similar sequences | None predicted | No GO terms |
| gene 13318 | *Cataglyphis hispanica* (LOC126853503) cover 40% identity 96.95% | None predicted | No GO terms |
| gene_14429 | *C. hispanica* (LOC126856133), cover 63% identity 95.28% | Spermadhesin (IPR035914), CUB domain profile (IPR000859), Ovochymase related (PTHR24251) | No GO terms |
| MB | | | |
| gene_3426 | *Formica exsecta* (LOC115243674), ncRNA cover 54% identity 87.42 % | Chitin binding peritrophin-A domain (IPR002557) | chitin binding (GO:0008061), extracellular region (GO:0005576) |
| gene_1607 | *C. hispanica* pheromone-binding protein Gp-9-like (LOC126850364), mRNA cover 33%, 93.58% identity, | Pheromone/general odorant binding protein (IPR006170), Pheromone-binding protein Gp-9 (IPR022354) | social behavior (GO:0035176), odorant binding (GO:0005549), extracellular space (GO:0005615) |
| gene_1243 | *C. hispanica* (LOC126849078), cover 25% identity 97.67%, | Prokaryotic membrane lipoprotein lipid attachment site profile (PS51257). | No GO terms |

Journal of Experimental Biology • Supplementary information

| | | | |
|---|---|---|---|
| gene_5298 | *C. hispanica* (LOC126851418), cover 19%, identity 86.15% | Pheromone/general odorant binding protein (IPR006170), PBP/GOBP family (PF01395) | odorant binding (GO:0005549) |
| gene_9900 | *C. hispanica* protein eiger (LOC126856729), cover 52% identity 97.89% | Tumor Necrosis Factor Ligand Superfamily (IPR051748), Protein Eiger (PTHR15151) | immune response (GO:0006955), tumor necrosis factor receptor binding (GO:0005164), protein binding (GO:0005515), membrane (GO:0016020), receptor ligand activity (GO:0048018) extracellular space (GO:0005615) |
| gene_7997 | *Pseudomyrmex gracilis* glycerate kinase-like (LOC109852192), cover 7%, identity 87.10% | None predicted | No GO terms |
| OL | | | |
| gene_5301 | *C. hispanica* pheromone-binding protein Gp-9-like (LOC126851279), cover 19%, identity 97.33% | Pheromone/general odorant binding protein (IPR006170), Pheromone-binding protein Gp-9 (IPR022354), PBP/GOBP family (PF01395) | social behavior (GO:0035176), odorant binding (GO:0005549), extracellular space (GO:0005615) |

Journal of Experimental Biology • Supplementary information

**Dataset 1. Raw data and results of "Transcriptional predictors of rescue behaviour in ants".** The Excel file includes 1) raw data of worker identity and colony, rescue behavior and brain size, 2) raw data of worker identity and colony, rescue behavior and morphological traits, 3) Tracked ant workers, general data from AnimalTA. Rescue behaviour yes or no. Neither no refer to ants that were too far from target, 4) Spatial data on tracked workers (inner and outer circle) from AnimalTA, 5) Results of tracked ant workers comparing high and low resolution from AnimalTA, 6) Differentially expressed genes (DEGs) for rescue behavior across all tissues, 7) Differentially expressed genes (DEGs) for rescue behavior in the optic lobes, 8) Differentially expressed genes (DEGs) for rescue behavior in the mushroom bodies, 9) Differentially expressed genes (DEGs) for rescue behavior in the central complex, and 10) Differentially expressed genes (DEGs) for rescue behavior in the antennae.

Available for download at
https://journals.biologists.com/jeb/article-lookup/doi/10.1242/jeb.252086#supplementary-data
